# Supplementary material for: Nanostructure ITO and Get More of It. Better Performance at Lower Cost
Source: Nanomaterials (Basel). 2020 Oct 5;10(10):1974. doi: 10.3390/nano10101974 (PMC7600850; doi:10.3390/nano10101974)
Supplement: Supplementary file 1 [file nanomaterials-10-01974-s001.pdf]

nanomaterials

Supplementary Materials

Nanostructure ITO and Get More of It. Better Performance at Lower Cost

Manel López [1,*], Juan Luis Frieiro [1], Miquel Nuez-Martínez [2], Martí Pedemonte [1], Francisco Palacio [1] and Francesc Teixidor [2,*]

[1] Department of Electronics and Biomedical Engineering, Faculty of Physics, University of Barcelona, Martí i Franquès street 1, 08028 Barcelona, Spain; jfrieiro@el.ub.edu (J.L.F.); pedemonte96@gmail.com (M.P.); fpalacio@el.ub.edu (F.P.)

[2] Institute of Materials , ICMAB-CSIC, Campus UAB, 08193 Bellaterra, Spain; mnuez@icmab.es

* Correspondence: manel.lopez@ub.edu (M.L.d.M.); teixidor@icmab.es (F.T.); Tel: +34-93-402-1147 (M.L.d.M.); Tel: +34-935-801-853 (F.T.)

1. SEM images and EDS results of the nanostructured electrodes prepared at several substrate temperatures and different times

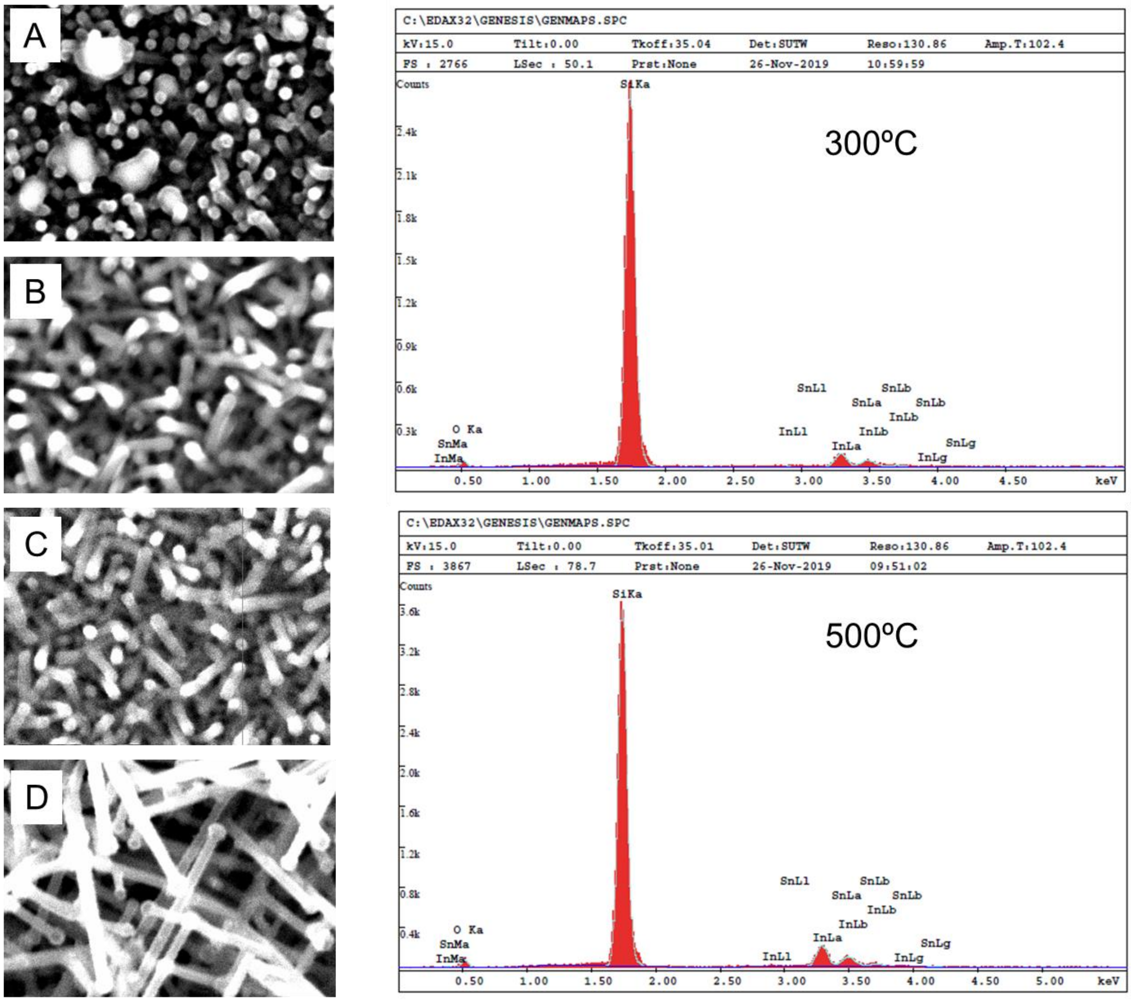

Figure S1. SEM images of nanostructured ITO electrodes prepared by electron beam evaporation, with substrate temperatures of (**A**) 200 °C, (**B**) 300 °C, (**C**) 400 °C and (**D**) 500 °C. EDS spectra obtained for nanostructured ITO electrodes at 300 °C and 500 °C of substrate temperature. All the samples were annealed at 600 °C, 1 h.

Figure S1 shows the evolution of the ITO nanowires for different growth temperatures. The self-catalytic VLS growth occurs from "seeds" that constitute regions offering high probabilities for the material to accumulate, inducing the nanostructuration appreciated in the SEM micrographs. The general mechanism for this growth can be explained by considering the binary phase diagram of the In-Sn eutectic system. Temperatures above ~120 °C permit the formation of In-Sn alloy droplets. Nanowire growth occurs by supersaturation of the catalyst drops with the vapor phase material, resulting in the growth of the crystalline nanowire: the material in vapor phase is dissolved in the drop and the crystallization occurs at the drop-material interface [1]. A proof of the self-catalytic VLS growth method is observed from the images shown in Figure S1. Indeed, if we carefully observe the shape of the nanowires and the tip of them, we observe that the seeds are present at that tip of the whisker. This is indicative of the growth process for the nanostructures. Other authors presented deep compositional studies showing by transmission electron microscopy (TEM) the composition and crystallinity of the nanowires and the morphological evolution [2], showing similar results to those presented in this paper.

Finally, the EDS spectra obtained for 300 °C and 500 °C show the same partial concentration of In and Sn, i.e. concentration ratio of In_2O_3:SnO_2 of 90:10 %wt. No changes were detected neither to the growth process nor the annealing at 600 °C 1h.

1. A. G. Nastovjak, I. G. Neizvestny and N. L. Shwartz, *Pure Appl. Chem.*, 2010, **82**, 2017–2025.
2. R. R. Kumar, K. N. Rao, K. Rajanna and A. R. Phani, *Mater. Res. Bull.*, 2014, **52**, 167–176.

2. Transmitance spectra of the ITO samples

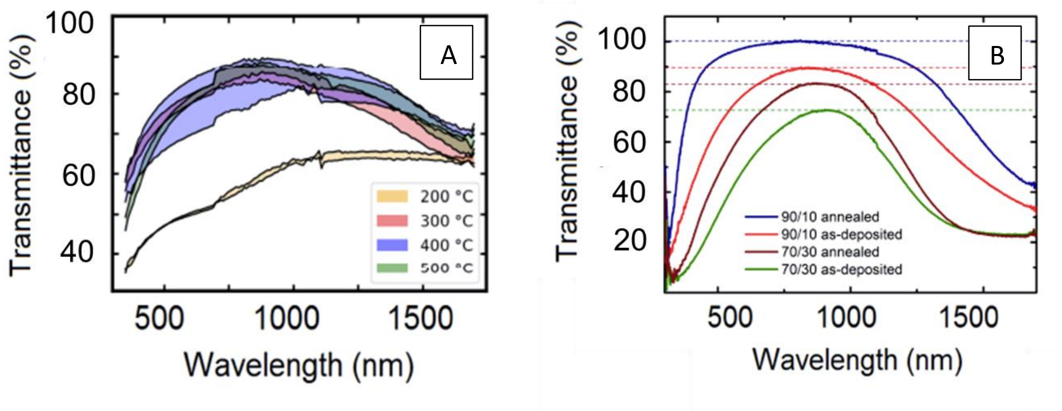

Figure S2. (**A**) Transmittance spectra for ITO nanowires as a function of the deposition temperature, for a constant concentration ratio of In_2O_3 : SnO_2 (90:10 wt%) and deposition time (30 min). (**B**) Transmittance spectra or ITO nanowires as a function of the concentration ratio. The concentration ratio of In_2O_3:SnO_2 varies from 70:30 to 90:10 wt%. for a constant temperature (300 °C) and deposition time (30 min). Blue and brown lines were annealed at 600 °C 1 h.

3. Preliminary PXRD analysis of the samples

In the PXRD study of the samples made at 300 and 500 ºC we have observed only the 2θ signals corresponding to the Si wafer (c-Si), shown next which suggests, in the absence of other more complete studies, that the ITO generated is amorphous, that is to say that the nano-wires are in a metastable state.

Figure 3 shows the PXRD spectra of the ITO samples grown at 300 and 500ºC with a further annealing at 600ºC 1h. As a comparison we include Figure S4. The XRD spectrum of Si crystalline wafers obtained from https://rruff.info/silicon/display=default/ on August 2020.

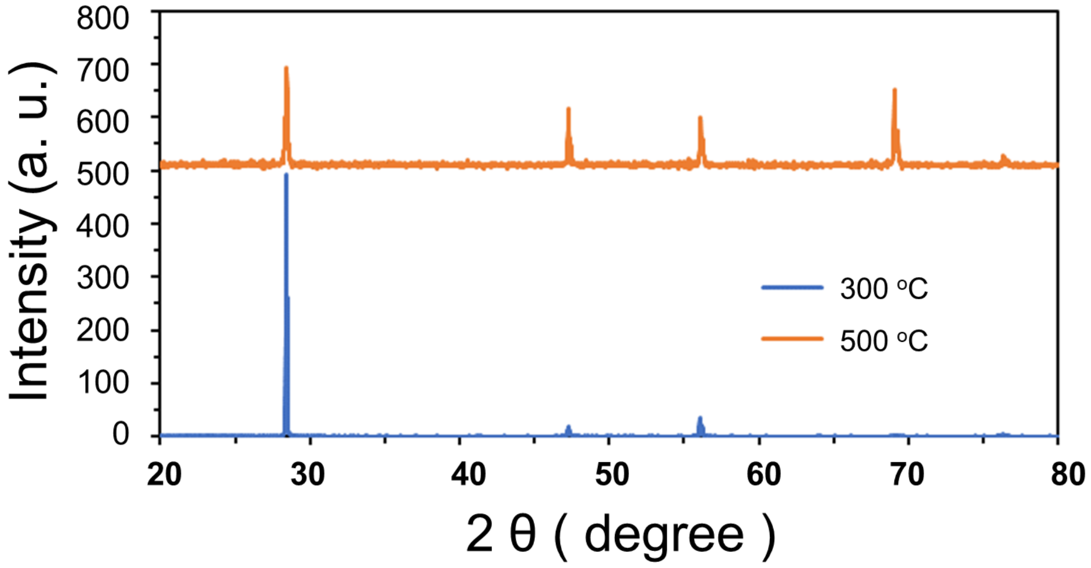

Figure S3. PXRD images of nanostructured ITO electrodes grown by electron beam evaporation on a Si wafer substrate. The process was done at substrate temperatures 300 °C, (blue line) and 500 °C (orange line). Samples were annealed at 600 °C 1 h.

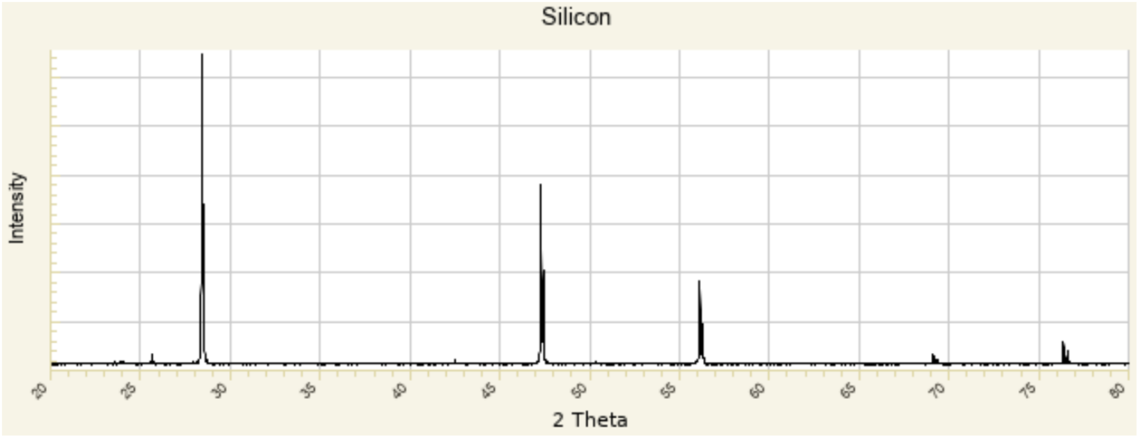

Figure S4. XRD images of Si crystalline wafers obtained from [3].

3. https://rruff.info/silicon/display=default accessed on 2020
